# Developing a Digital-Based Exercise Snack Program for Community-Dwelling Frail Older Adults

**DOI:** 10.1093/geroni/igaf122.2825

**Published:** 2025-12-31

**Authors:** Min Kyung Park, Gwang Suk Kim, Jae Jun Lee, Layoung Kim, Sooyoung Kwon, Eun Ju Park, Hayejin Yang, Seungbum Yang

**Affiliations:** Yonsei University College of Nursing, Seoul, Republic of Korea; Yonsei University College of Nursing, Seoul, Republic of Korea; Yonsei University College of Nursing, Seoul, Republic of Korea; The University of Suwon, Hwaseong, Republic of Korea; Graduate School of Yonsei University, Seoul, Republic of Korea; Yonsei University College of Nursing, Seoul, Republic of Korea; Graduate School of Yonsei University, Seoul, Republic of Korea; Graduate School of Yonsei University, Seoul, Republic of Korea

## Abstract

Frail older adults’ physical limitations hinder their engagement in traditional continuous exercise or group-based physical activities. A digital exercise snack program, comprising short, frequent exercises at home, may be a viable alternative. This study aimed to develop and evaluate the feasibility and acceptability of such a program. The program was developed in four stages: (1) initial design, (2) content validation by six experts, (3) refinement and a one-week pilot with the research team, and (4) a two-week pilot with three frail older adults assessing the feasibility (completion, safety, and adherence) and usability of the digital device. The item-content validity index of the program’s preliminary version ranged from 0.67 to 1.00. The final program included five exercises (arm curl, marching on the spot, sideways leg lift, sit-to-stand, and calf raise), each followed by a one-minute grip ball rest, totalling ten minutes per session. Participants followed the program twice daily in the first week and thrice daily in the second week at their preferred time, with the digital device providing guidance, adherence monitoring, personalized reminders, session recordings, and pop-up notifications. All participants completed the two-week program, with no safety issues above CATAE grade 3 reported. On average, participants performed 29.3 exercise sessions, with an adherence rate of 97.4%. Digital device usability and program satisfaction were assessed on a 5-point scale, with average scores of 4.3 and 4.7, respectively. The program demonstrated adequate feasibility and adherence, highlighting its potential in facilitating physical activity among frail older adults.

